# Impact of entrepreneur’s gender on innovation activities. The perspective of small businesses

**DOI:** 10.1371/journal.pone.0258661

**Published:** 2021-10-20

**Authors:** Maciej Zastempowski, Szymon Cyfert

**Affiliations:** 1 Faculty of Economic Sciences and Management, Department of Enterprise Management, Nicolaus Copernicus University, Torun, Poland; 2 Institute of Management, Poznan University of Economics and Business, Poznan, Poland; Politecnico di Torino, ITALY

## Abstract

This paper analyses the female gender as a one of the factors that may influence product and process innovativeness of small enterprises. The data discussed come from an empirical study of 1017 small enterprises from the Kujawsko-pomorskie region in central-northern Poland. The theoretical framework suggests three areas of factors that may influence small enterprises innovativeness: the entrepreneur’s gender, innovation management capability, and firm characteristics. The results of the study suggest that the female gender of the entrepreneur has a positive impact on the product and process innovativeness of small enterprises. In the group of small enterprises managed by female entrepreneurs the chances of introducing product innovation are higher by 83.7%, process innovation by 56%, and product and process innovation together by 82.1%.

## 1. Introduction

The question of how to create innovation in companies is one of those questions that we are still looking for answers to [1–3]. Although innovation is central to improvements in living standards and can affect individuals, institutions, entire economic sectors, and countries in multiple ways [4], still many areas of research on it constitute a kind of a white spot. One of them is the innovativeness of small enterprises. Here, one can still point to the existing knowledge gaps, especially in the area of internal factors that can stimulate innovativeness of small enterprises [5–9]. One of such factors that receives scarce attention is the gender of the enterprise owner and/or manager [10], considered as one of the elements of management capabilities [11]. The purpose of this text is to try to shed additional light on this interesting issue.

In terms of the impact on the development of research on the innovation issues, and primarily the interdependence of the development of science, technology and economy, the role of the protagonist is played by Schumpeter [12]. Since when he indicated the entrepreneur as a person whose main function is to make a new combination of production factors, innovation has been treated as a key dimension of entrepreneurship [13]. Consequently, it is one of the criteria by means of which we distinguish a true entrepreneur from ordinary business owners [14] or leaders from followers [15]. In turn, small entrepreneurial firms, in line with the concept of creative destruction, are at the centre of the innovation process [16]. Despite this perspective, research on innovation in the context of small entrepreneurial firms is still limited [17]. It is also worth emphasizing that there are big differences in how small businesses are defined. For example, taking only the employment criterion into account, in the United States a small company in the manufacturing sector employs less than 500 employees, and in the wholesale trade sector—less than 100 [18], while in the European Union less than 50 employees [19]. This article deals with the European perspective of a small business as a company employing from 10 up to 49 employees.

One of the important aspects of research on small business innovation is its innovation capability [20–26]. Usually, from the Resource-Based View, it is understood as a combination of various types of resources conducive to the emergence of innovation [27,28]. It is also indicated that innovation capability allows the organization to adopt to competition, the market and environment [29,30]. Despite a number of studies, the question of what elements ought to create innovation capability so as to effectively introduce innovations is still valid. One such element worth researching, also from the perspective of Upper Echelons Theory [31,32], are the characteristics of a small business owner [33,34], including the gender identity. This seems particularly important because, on the one hand, most research on innovation ignores the gender issue of the innovator [35,36], and on the other hand, however, they often focus on the analysis of industries that are male-dominated and embody a masculine perspective [37,38].

Although some studies devoted to the relationship between female entrepreneurship and innovation suggest no significant differences in innovation between male and female entrepreneurs [39], most studies notice these differences [36,40–43]. However, these studies ignore the impact of enterprise size and there a few research relating to the innovativeness of women in small and medium-sized enterprises (SMEs) [43,44], although the results of previous studies suggest that the characteristics of SMEs mean that innovative activities of SMEs differ from innovative activities undertaken in large enterprises [26,45–49]. Our article has a contribution to the literature on the role of women in implementing innovative activities in three dimensions. First, we focus on the impact of the entrepreneur’s gender on innovation activities from the perspective of small enterprises. Second, Arun and Rojers suggest empirical investigations are primarily influenced by literature from the Anglo-Saxon areas [50], while we look at the relationship between gender and innovation activities from the Polish perspective, which strengthens the arguments for the existence of an entrepreneur’s gender influence on innovation activities. Third, we research the influence of female entrepreneurs on the innovativeness of small enterprises based on data from a survey of 1017 small firms from the kujawsko-pomorskie region in central-northern Poland, referring to the general management capabilities of a firm. Besides contributing to the literature, the results of our research have value in taking action in the areas of promoting innovation and equal opportunities for women and men. The article is structured as follows: in Section 2, we present the theoretical framework, focusing on innovation management capability, gender of entrepreneur and organization characteristics. We also indicate the proposed theoretical model along with our hypotheses. In Section 3, we discuss the empirical research conducted, indicating the method of obtaining data, characterizing the research sample, the adopted variables and the logit regression models used. Section 4 presents the estimation results of three logit regression models. Section 5 contains a discussion of these results. The paper finishes with conclusions in section 6.

## 2. Literature review and conceptual framework

Akman and Yilmaz believe that innovation could be recognized as a key success factor in an increasingly competitive, global economy [51]. In order to be able to conduct research in the field of innovation, innovation needs to be defined. However, a problem arises here—the problem of the ambiguity of the word ‘innovation’. It can be perceived from several different perspectives: (1) process—as an innovative process with a specific beginning and end [13,52–55]; (2) attributes—as a set of features (organization or unit) generating the ability and propensity to innovate [56,57]; (3) result—as a result of the innovative process, *i*.*e*., the implementation of innovation [58,59]. The same applies to other concepts related to innovation. In this text, we understand innovation as the result (effect), *i*.*e*., the implementation of innovation. Sharing the opinion of Oslo Manual that innovation is more than a new idea or an invention and an innovation requires implementation (either by being put into active use or by being made available for use by other parties, firms, individuals or organizations), we define it as follows–‘an innovation is a new or improved product or process (or combination thereof) that differs significantly from the unit’s previous products or processes and that has been made available to potential users (product) or brought into use by the unit (process)’[11].

Research on the issue of innovation that has been conducted for many years attempts to identify various factors that may affect it. Both corporations [30,60,61] and smaller enterprises [21,62–65], as well as micro-entrepreneurs and self-employed [34,66]. Their analysis allows to indicate several areas that may turn out to be important from the point of view of innovativeness of small enterprises. These are mainly innovation management capability and company characteristics. From the perspective of the purpose of this text, we also want to draw attention to the gender of the entrepreneur.

### 2.1. Innovation management capability

Many works in subject literature are devoted to the analysis of innovation, and especially to innovation capabilities [67,68]. As suggested by Guan and Ma, innovation capabilities are usually understood as a combination of various types of resources favouring the emergence of innovation [30]. Martinez-Roman et al. point to three key dimensions of innovation capability: knowledge, organization, and human factor [65]. In turn, Yam et al. suggest a division into seven elements: learning capability, R&D capability, resource allocation capability, manufacturing capability, marketing capability, organization capability and strategic planning capability [69]. The approach resulting from the Oslo Manual is also interesting and encouraging research. In line with it, numerous business capabilities can potentially support innovation activities and the economic success of innovations. This methodology identifies four types of capabilities that are important form the perspective of research on the innovation performance of firms: (1) the resources controlled by a firm, (2) the general management capabilities of a firm, including capabilities related to managing innovation activities, (3) the skills of the workforce and how a firm manages its human capital, and (4) the ability to develop and use technological tools and data resources, with the latter providing an increasingly important source of information for innovation [11]. Taking into account the perspective of the entrepreneur’s gender, we would like to focus on the second type of capabilities mentioned in this methodology, *i*.*e*., the general management capabilities of a firm. This kind of capabilities can influence a firm’s ability to undertake innovation activities, introduce innovations and generate innovation outcomes. They include (1) business strategy, (2) organisational and managerial capabilities, (3) characteristics of the business owner and top management, (4) innovation management capabilities and (5) intellectual property management and appropriation [11].

Taking into account the purpose of this text, we will analyse the characteristics of the business owner and top management (*e*.*g*., sex or gender identity) and innovation management capabilities in the following part of the detailed analysis, consisting of the following seven types of abilities: (1) identifying, generating, assessing, and pursuing ideas for innovation, (2) organizing innovation activities within the firm, (3) allocating resources to innovation activities, (4) managing innovation activities conducted in collaboration with external partners, (5) integrating external knowledge and other external inputs into a firm’s innovation activities, (6) monitoring the results of innovation activities and learning from experience, and (7) exploiting and managing innovations and other knowledge that has been generated as part of a firm’s innovation activities [11].

#### 2.1.1. Identifying, generating, assessing, and pursuing ideas for innovation

Abilities in the area of identifying, generating, assessing and pursuing ideas for innovation constitute the foundation of the process of shaping innovation. At this stage, innovation opportunities are assessed and decisions are made in relation to the involvement of organizational resources in innovation activities [70]. In the context of the activities carried out at the stage of identifying, generating, assessing, and pursuing ideas for innovation Neneh emphasizes the role of SME owners and managers in identifying, developing and marketing new products and processes [46]. However, the results of the studies by Nählinder et al. suggest that there is no significant difference in innovation between men and women, the researchers point to the need to correct the gender-dominant approach in research on innovation, because in the processes of identifying and assessing ideas for innovation, women are more likely to choose less risky options than men [39]. Also, the results of the research performed by Reutzel suggest that women-managed enterprises, due to their perceived lower environmental goodness and distributional equity, have a lower level of investment in innovation than men-led enterprises [71]. Similarly, conclusions pointing to the complexity of the relationship between organizational skills, organizational culture and gender are formulated by Cropley and Cropley who claim there is no influence of gender balance on the emergence of incentives for innovation [40]. In turn, Jabeen et al. when conducting research on the functioning of SMEs in the Emirates [72] noted that female owners treat research and development, innovation strategy and skills development as the major criteria in decisions related to innovation. Foss et al. suggest that although women are characterized by a similar level of innovation in generating new ideas as men, their level of innovation activity is lower, as women’s ideas are less frequently implemented in the organization. Generating and implementing ideas by women in male-dominated organizations may be limited by organizational practices or by the perceived by women a lower level of support obtained from superiors [73]. Díaz-García et al. suggest that gender diversity within R&D teams generates certain dynamics that foster novel solutions leading to radical innovation [74].

#### 2.1.2. Organising innovation activities within the firm

Before company managers can concentrate on activities related to the development and commercialization of innovations, they must define the way of organizing the innovation process. When referring to the activities carried out at this stage, Bergfors and Larsson emphasize the need to adjust the strategy and structure internally, as well as the importance of choices made by managers regarding the centralization or decentralization of product and process innovations. At the same time, they suggest that R&D activities that are strategically important or that target radical innovation should be centralized, while R&D activities that are less strategically important and that are geared towards incremental innovation should be decentralized [75]. The size of the enterprise plays an important role in the organization of innovative activity—Corsi et al. indicate that while small enterprises characterized by a high level of flexibility relatively easily adapt innovations to introducing organizational changes in order to improve their effectiveness, large enterprises face resistance related to the acceptance and integration of innovations [76]. However, it is worth noting that high-tech SMEs engage more in product development and process innovation, whereas non-high-tech SMEs tend to associate more in organizational and market innovations [77]. A similar observation is made by Hirsch-Kreinsen, pointing out that SMEs not related to advanced technologies limit their own R&D activity, but given the limited formalization of knowledge creation and use, innovative activities usually take the form of application-oriented practical knowledge Hirsch-Kreinsen [45]. The results of the studies by Foss et al. suggest that centralization has a negative impact on the implementation of new ideas by employees, with this effect being gender-moderated and stronger for women than men [78]. Also, Kvande and Rasmussen’s research on gender in organizations indicates the advisability of reducing hierarchy and bureaucracy [79]. A similar conclusion is reached by Fenwick who suggests that women seek challenges and diversity, and even crises, in their daily work as a means of stimulating innovative learning [80]. In turn, the preliminary results of the Cropley and Cropley study suggest a negative relationship between the percentage of women in functional areas and the ability to innovate, with the researchers pointing out, similar to Foss et al. [78] that women’s ability to innovate is limited by an unfavourable organizational climate [40]. Explaining the differences between innovation in terms of gender, Brusch et al. indicate that female entrepreneurs face greater domestic burdens due to which tend to locate their businesses closer to home; female entrepreneurial ventures are thus less likely to be found in highly interactive, innovative, and productive agglomerations of economic activity [81].

#### 2.1.3. Allocating resources to innovation activities

Outcome of the research suggests that innovative processes require managers to use the ability to properly allocate and share activities and resources within the enterprise [82]. This means that the effectiveness of innovation depends on proper management of intangible resources and coordination of activities in the organization [83,84]. Ayalew and Xianzhi indicate the negative impact of financial constraints on companies’ decisions regarding involvement in innovative activities [85]. Turning to research on women’s risk aversion, Nählinder suggests that female-led companies pursue investment strategies that are less risky than male-led companies, and that female-led companies invest less resources in innovation than companies by men [86]. A similar phenomenon is emphasised by Reutzel et al., who refer to studies in the field of psychology suggesting that gender may influence the willingness to invest, and indicate that women are reluctant to engage in risky activities when compared to men [71].

#### 2.1.4. Managing innovation activities conducted in collaboration with external partners

Justifying the advisability of abilities connected with managing innovation activities conducted in collaboration with external partners, Expósito points out that in the global world individual enterprises cannot afford to fully rely on their own research and resources [87]. Wynarczyk also raises the issue of the deficit of internal resources, which makes it difficult for SMEs to master new factors of production, innovation and competitiveness [48]. Under such conditions, building relational capital and promoting positive relations with internal and external stakeholders become important components of innovative strategies that help to shape a competitive advantage. Neneh suggests that while the customer focus is a valuable in-house strategic capability, its performance benefits for SMEs may be limited as SMEs are unable to respond quickly to customer needs due to limited resources. SMEs are trying to reduce this problem by relying on their business connections and social networks for valuable resources to effectively harness the opportunities arising from identified customer needs [82]. Carrasco, in turn, highlights the importance of networks and communities for [88]. Although the results of the study by suggest that women, as business owners, are more likely to cooperate in networks than men [89,90], Reutzel et al. suggests that female-led companies perceive less environmental goodness and distribution fairness [71].

#### 2.1.5. Integrating external knowledge and other external inputs into a firm’s innovation activities

Akram et al. indicate that firms no longer rely on just in-house knowledge generation for carrying out innovation within their organizational boundaries but they recognize the importance of external knowledge search for innovation [91]. A particularly important abilities are connected with a process of integrating external knowledge and other external inputs into a firm’s innovation activities can be found in the case of SMEs due to limited resources [47], which makes it necessary to develop mechanisms that allow for acquiring external knowledge, transfer it internally and integrate with existing resources [92]. By pointing out that many small businesses lack the knowledge, time, and resources to innovate, Martin argues that companies that can use their internal resources and enhance them by linking to external support sources have a competitive advantage in terms of innovation [44]. Subrahmanya, in turn, draws attention to the importance of acquiring external knowledge and skills, allowing them to supplement their own competences in this way [49]. Nählinder points out that female entrepreneurs, when trying to access resources from their environment in search of innovative opportunities, have to face various problems, which is why they perceive the business environment as less favourable than men do. They have more limited access to human and social capital related to innovation, and, moreover, they start ventures with fewer resources and are less familiar with how to obtain external finance capital [86].

#### 2.1.6. Monitoring the results of innovation activities and learning from experience

Rowley emphasizes that organizations that strive for the status of knowledge-based entrepreneurs must go beyond ‘knowledge management’ and must shape the connections between people and systems [93], which in the case of abilities referred to monitoring the results of innovation activities and learning from experience become a key element. However, the results of Michna’s research suggest the existence of a relationship between learning and performance [94]. When conducting research on knowledge management, Martin indicated that female managers and their employees perceive knowledge as an asset and develop organizational values supporting the creation of knowledge, emphasizing dialogue as a key element of communication [95].

#### 2.1.7. Exploiting and managing innovations and other knowledge that has been generated as part of a firm’s innovation activities, including protecting knowledge and innovation assets

Cropley and Cropley indicate that the better the innovation capacity is built, the more effectively the enterprise can implement the innovation process, and thus it can achieve better results in terms of innovation [40]. Ruiz suggests that because gender diversity entails additional heterogeneity related to gender-related experience, knowledge and skills, gender diversity influences the way organizations connect and use knowledge to generate innovation. Moreover, gender diversity can contribute to achieving greater complementarity between members of the management team, compensating the weaknesses of some members with the strengths of others, and enhancing the ability to combine knowledge to generate new innovations [63]. In turn, the results of the study by Foss et al. suggest the need to pay more attention to women’s innovation in male-dominated corporations [78]. The results of the Fenwick research show that the strong preference for spontaneity by women means that both long-term processes of institutionalization and structures supporting strategic learning may seriously limit and discourage innovative energy sources [96].

### 2.2. The entrepreneur’s gender

Treating the Upper Eschelon Theory as a starting point, *i*.*e*., the theory according to which organizational outcomes are partially predicted by managerial background characteristics of the top-level management team [97], it is worth considering whether the gender of the entrepreneur also plays such a role. Sharing the opinion of Alsos et al. according to which most research in the field of innovation ignores the gender issue of the innovator [35] and of Henry et al.—they focus on the masculine perspective [37], it is worth noting that only a few studies seem to confirm the important role of women as entrepreneurs, owners or managers in the process of creating innovation. This is suggested by research by Roper et al. [66], Chen and Yang [98], Lin [99], Horbach and Jacob [100], Ruiz-Jaménez et al. [63], Na and Shin [101], Torchia et al. [102] or Dohse et. al. [103]. As a consequence, we decided to examine the influence of a female entrepreneur behaviour on innovativeness of a small enterprise.

### 2.3. Organization characteristics

When trying to present the characteristics of enterprises used in research on innovation, which are important for its identification, it should be emphasized that the most frequently used ones include the company’s age [104–106] and its size [24,30,107–109]. It can be said that they are genetic by nature. Their impact on innovation has been described many times, especially in terms of company size [12,110,111]. Therefore, we decided to use them in our research.

### 2.4. Conceptual model and hypotheses

**Fig 1:**
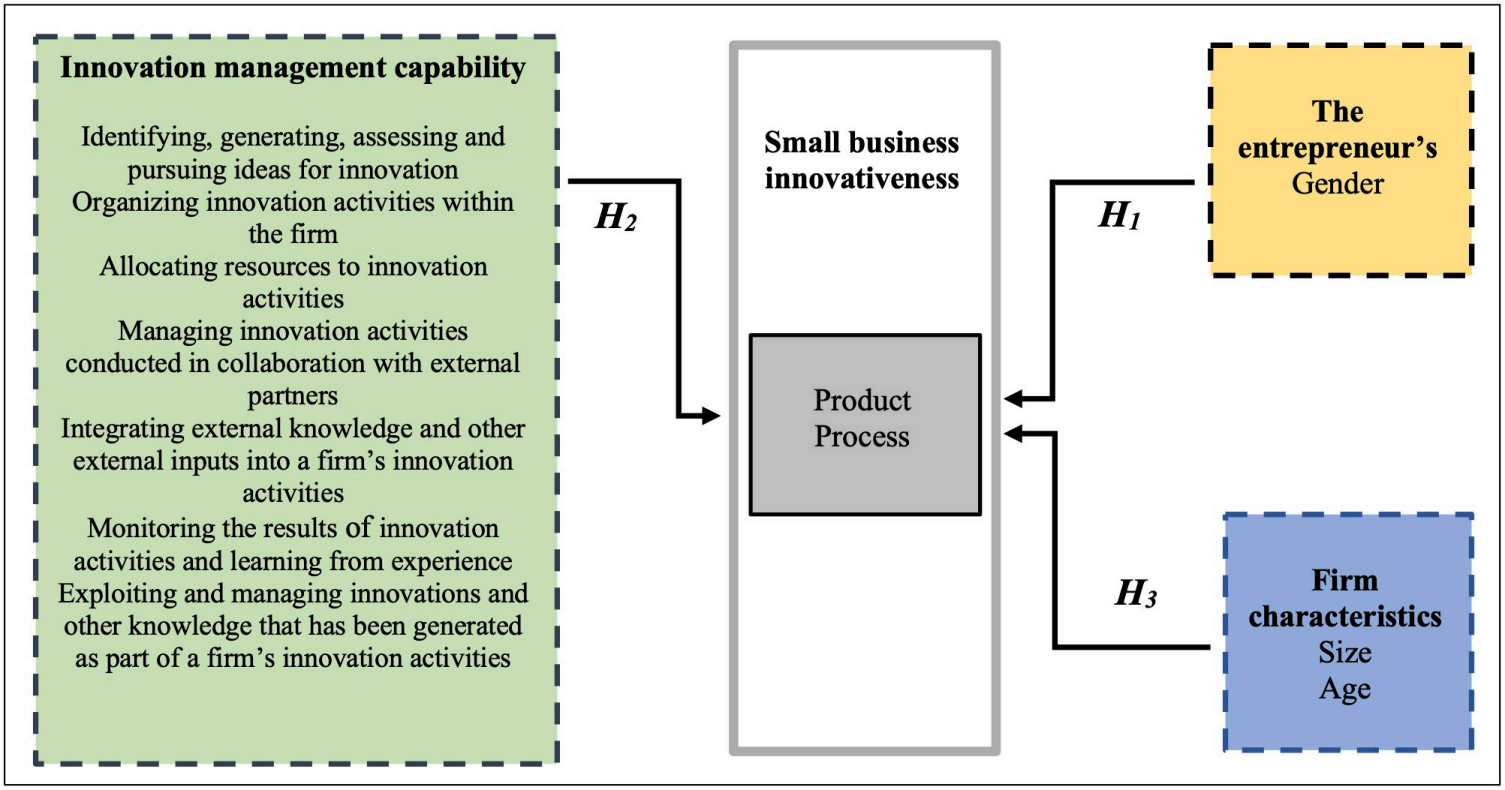
The theoretical model of the study.

Based on the presented literature review, we can identify three basic dimensions which, together with the factors assigned to them, allow us to construct a theoretical model (Fig 1).

As can be seen, the theoretical model includes three categories of explanatory variables: the entrepreneur’s gender, innovation management capability, and firm characteristics; and one category of dependent variables: small business innovativeness (product and process innovations). Consequently, we propose the following three hypotheses:

H_1_—The entrepreneur’s female gender exerts a positive impact on small business innovativeness.
H_2_—Innovation management capability exerts a positive impact on small business innovativeness.
H_3_—Firm characteristics exert a positive impact on small business innovativeness.

## 3. Materials and methods

### 3.1. Data collection

The data presented in this study come from empirical research conducted from June to September 2019, as a part of the research project called Regiogmina, which the main goal was to prepare a reliable and up-to-date picture of competitiveness, innovativeness and cooperation among SMEs in the Kujawsko-pomorskie region in central-northern Poland. The Computer Assisted Personal Interview (CAPI) method was used. The firms participating in the survey were randomly selected by the Kujawsko-pomorskie Statistical Office, based on the REGON register (The National Official Register of Economy Entities). The stratified sample—with quotas for sectors (according to Code List of Classification of Business Activities in Poland—PKD 2007), subregion (NUTS 3) and county (NUTS 4)–was representative for the population of small enterprises in the Kujawsko-pomorskie region (6,732 small enterprises in 2019) [112] with an error of +/- 2% at a confidence level of 99%. The final dataset is made up by of 1017 observations from small enterprises.

### 3.2. Sample

As can be seen in Table 1, the sample examined represented all types of economic activity. Only two of them had a higher representation than in REGON: manufacturing (13.8%) and wholesale and retail trade (5.8%), and one had lower representation, *i*.*e*., transport and storage (4.1%).

**Table 1:** Structure of the sample.

Activities (PKD 2007^b^)	REGON^a^ (%)	Sample (%)	Difference: REGON—Sample (% point)
A—agriculture, forestry, hunting and fishing	2.0	1.9	0.1
B—mining and quarrying	0.1	0.1	0.0
C—manufacturing	8.8	22.6	-13.8
D—electricity, gas, steam, hot water and air conditioning	0.3	0.2	0.1
E—water supply; sewage and waste management and remediation activities	0.4	1.5	-1.1
F—building construction	13.5	10.7	2.8
G—wholesale and retail trade; repair of motor vehicles, excluding motorcycles	22.5	28.3	-5.8
H—transport and storage	6.7	2.6	4.1
I—activities related to accommodation and catering services	2.4	3.5	-1.1
J—information and communication	2.6	2.7	-0.1
K—financial and insurance business	3.0	1.8	1.2
L—activities related to the real estate	5.2	4.6	0.6
M—professional, scientific and technical activity	8.4	6.3	2.1
N—administration and support activities	3.1	2.8	0.3
O—public administration and defence; mandatory social security	0.7	0.5	0.2
P—education	3.4	0.9	2.5
Q—health care and social welfare	7.6	4.4	3.2
R—activities related to culture, entertainment and recreation	2.1	0.6	1.5
S—other service activities	7.2	4.0	3.2

^a^ REGON—National Official Register of Economy Entities is the official Poland’s registry kept by the Central Statistical Office in Poland.

^b^ PKD 2007—The Code List of Classification of Business Activities in Poland.

### 3.3. Variables

The following explained and explanatory variables were introduced into the logistic regression models.

The explained variables are the results of small business innovativeness. Small firm owners and managers were asked, whether in the previous three years (2016, 2017 and 2018) they had introduced a new or improved product or process (or a combination thereof) that differs significantly from the unit’s previous products or processes and that has been made available to potential users (product) or brought into use by the unit (process). As a consequence, the three dummy variables were used to describe the results of small business innovation activities:

1. *Product innovation* (y_1_)—this variable takes the value 1 if small firm introduced a new or improved product in the previous three years, and the value 0 if not;
2. *Process innovation* (y_2_)—this variable takes the value 1 if small firm introduced a new or improved business process in the previous three years, and the value 0 if not;
3. *Product and process innovation* (y_3_)—this variable takes the value 1 if the small firm introduced both a new or improved product and process in the previous three years, and the value 0 if not.

The explanatory variables were divided into three groups:

1. Innovation management capability—indicators for innovation management capability ware measured on an ordinal scale, and take values from 1 to 7, where 1 means complete disagreement and 7 full agreement:
  - *Identifying*, *generating*, *assessing and pursuing ideas for innovation* (x_1_),
  - *Organizing innovation activities within the firm*—(x_2_),
  - *Allocating resources to innovation activities*—(x_3_),
  - *Managing innovation activities conducted in collaboration with external partners*—(x_4_),
  - *Integrating external knowledge and other external inputs into a firm’s innovation activities*—(x_5_),
  - *Monitoring the results of innovation activities and learning from experience*—(x_6_),
  - *Exploiting and managing innovations and other knowledge that has been generated as part of a firm’s innovation activities*—(x_7_);
2. Firm characteristics:
  - *Size* (x_8_)—firm size measured by the number of employees—this variable is numerical and a logarithm was applied for the calculations;
  - *Age* (x_9_)—age of the firm measured by the number of years since the business was founded—this variable is numerical and a logarithm was applied for the calculations;
3. The entrepreneur’s gender:
  - *Female* (x_10_)—this variable takes the value 1 if the small firm entrepreneur was female, and the value 0 if not.

A description and scale for all variables included in this research are presented in Table 2. In turn, the descriptive statistics are in Table 3.

**Table 2:** Description of variables.

Description	Label	Type
**EXPLANATORY VARIABLES**		
**Innovation management capability**		
Identifying, generating, assessing and pursuing ideas for innovation	x_1_	Ordinal (1–7)
Organizing innovation activities within the firm	x_2_	Ordinal (1–7)
Allocating resources to innovation activities	x_3_	Ordinal (1–7)
Managing innovation activities conducted in collaboration with external partners	x_4_	Ordinal (1–7)
Integrating external knowledge and other external inputs into a firm’s innovation activities	x_5_	Ordinal (1–7)
Monitoring the results of innovation activities and learning from experience	x_6_	Ordinal (1–7)
Exploiting and managing innovations and other knowledge that has been generated as part of a firm’s innovation activities	x_7_	Ordinal (1–7)
**Firm characteristics**		
Size—number of employees	x_8_	Numerical
Age—number of years since business founding	x_9_	Numerical
**The entrepreneur’s gender**		
Female	x_10_	Dichotomous
**EXPLAINED VARIABLES**		
Product innovation	y_1_	Dichotomous
Process innovation	y_2_	Dichotomous
Product and process innovation	y_3_	Dichotomous

**Table 3:** Descriptive statistics of variables.

Variables	Mean	S.E.	S.D.	SD^2^	Min.	Max.
y_1_	0.195	0.012	0.396	0.157	0	1
y_2_	0.156	0.011	0.363	0.132	0	1
y_3_	0.125	0.010	0.331	0.109	0	1
x_1_	3.741	0.059	1.868	3.491	1	7
x_2_	3.533	0.065	2.078	4.316	1	7
x_3_	2.261	0.054	1.729	2.990	1	7
x_4_	3.051	0.059	1.869	3.491	1	7
x_5_	4.395	0.042	1.349	1.820	1	7
x_6_	4.421	0.044	1.414	2.000	1	7
x_7_	4.398	0.042	1.335	1.781	1	7
x_8_	2.839	0.019	0.616	0.380	0	5.017
x_9_	2.981	0.014	0.444	0.198	2.303	3.892
x_10_	0.335	0.015	0.472	0.223	0	1

### 3.4. Methods

In this research we examined the group of small enterprises, however our research involved human participants, since we asked entrepreneurs or managers to answer the survey questions. All participants were informed that the survey is anonymous. We also analysed data anonymously, and we did not ask about any personal information. Therefore, our research, in accordance with the recommendations of the National Science Centre [113] which are the basis for drawing up guidelines for conducting research at our Universities [114,115] did not require approval of the ethics committees.

In order to investigate the influence of the explanatory variables on the dichotomous explained variable y_1_ (product innovation), y_2_ (process innovation) and y_3_ (product and process innovation) the logit regression model was used. It can be written as follows:

$$logit\;(p_{i})=Z_{i}=x_{i}^{\prime }\beta =\beta_{0}+\beta_{1}X_{1i}+\beta_{2}X_{2i}+\ldots +\beta_{k}X_{ki}$$ (1)

where we denoted $\text{ln}\frac{p_{i}}{1-p_{i}\;}$. as *logit* (*p*_*i*_). The subject of estimation in this model are the parameters *β*_0_, *β*_1_, *β*_2_, …, *β*_*k*_ being elements of the *β* vector [116].

For the interpretation of the results of the logit model estimation, we used odds ratios (OR). If we mark the chance as:

$$\frac{p_{i}}{1-\;p_{i}}=\text{exp}\left(x_{i}^{\prime }\beta )=exp(\beta_{0\;}+\beta_{1}X_{1i}+\beta_{2}X_{2i}+\ldots +\beta_{k}X_{ki}\right)=\Omega (x_{i})$$ (2)

is the odds ratios with the variable *X*_*mi*_ increased by a unit and the odds without this increase equal:

$$\frac{\Omega (x_{i}^{m},{\;X}_{mi}+1)}{\Omega (x_{i}^{m},\;{\;X}_{mi})}=exp(\beta_{m})$$ (3)

where $x_{i}^{m}$ is the vector *x*_*i*_ without the variable *X*_*mi*_. Formula (3) shows that the increase in the value of *X*_*mi*_ by one unit is related, *ceteris paribus*, with an *exp*(*β*_*m*_)-fold change in the odds ratio. In the case of *exp*(*β*_*m*_) > 1 we have an increase, and in the case of *exp*(*β*_*m*_) < 1 we have a decrease in the odds ratio. For example, if *X*_*m*_ is a binary variable, *exp*(*β*_*m*_) informs how many times the odds ratio of *Y*_*i*_ = 1 increases for the "1" category of the variable *X*_*m*_ as compared to the same odds ratio for the category "0" of the variable *X*_*m*_. The odds ratios are the values of $exp\left(\hat{\beta_{j}}\right)$, where $\hat{\beta_{j}}$ are the estimates of the parameters of the logit model. These are the folds by which the odds ratios change on average with the increase of each variable by a unit.

To estimate the models, we used the maximum likelihood estimation (MLE) method and the STATA.16.1 software.

## 4. Results

In the first step, we analysed the correlation between the variables included to the models. The results are presented in Table 4.

**Table 4:** Correlation matrix.

**Variables**	**y**_**1**_	**y**_**2**_	**y**_**3**_	**x**_**1**_	**x**_**2**_	**x**_**3**_	**x**_**4**_	**x**_**5**_	**x**_**6**_	**x**_**7**_	**x**_**8**_	**x**_**9**_	**x**_**10**_
y_1_	1.000												
y_2_	0.657**	1.000											
y_3_	0.768**	0.878**	1.000										
x_1_	0.246**	0.209**	0.246**	1.000									
x_2_	0.027	0.027	0.048	0.373**	1.000								
x_3_	0.071*	0.105**	0.103**	0.112**	0.338**	1.000							
x_4_	0.156**	0.103**	0.132**	0.432**	0.414**	0.329**	1.000						
x_5_	0.263**	0.232**	0.246**	0.077**	-0.176**	-0.170**	-0.076**	1.000					
x_6_	0.252**	0.224**	0.239**	0.191**	-0.065**	-0.142**	0.011	0.418**	1.000				
x_7_	0.268**	0.240**	0.259**	0.236**	-0.033	-0.163**	0.058*	0.374**	0.327**	1.000			
x_8_	0.011	0.005	0.013	-0.020	0.000	-0.040	-0.009	-0.003	-0.012	-0.024	1.000		
x_9_	-0.031	0.025	0.002	0.048*	0.191**	0.212**	0.129**	-0.093**	-0.081**	-0.049*	0.064**	1.000	
x_10_	0.119**	0.078*	0.091**	0.003	-0.130**	-0.168**	-0.106**	0.074**	0.142**	0.080**	-0.016	-0.110**	1.000

** p-Value ≤ 0.01.

* p-Value ≤ 0.05.

In Table 4 we can see, that some correlations coefficients among the explained and explanatory variables are statistically significant. The relationships are positive and negative. Nevertheless, the coefficients are always below 0.27, so the relationship is very poor. Also, the correlations coefficients among the explanatory variables (innovation management capability, firm characteristics and entrepreneur’s gender) are below 0.5 what indicating that multicollinearity in not a concern.

The results of the estimations are shown in Table 5—for product innovation, in Table 6—for process innovation, and in Table 7 –for both types of innovation together. In those tables, in column S.E, robust standard errors are presented.

**Table 5:** Logistic regression for product innovation.

Variables	Model 1		Model 2		Model 3		
	*β*	S.E.	*β*	S.E.	*β*	S.E.	OR
x_1_	0.298***	0.061	0.300***	0.061	0.299***	0.062	1.349***
x_2_	-0.085	0.057	-0.080	0.057	-0.071	0.057	0.932
x_3_	0.191***	0.056	0.202***	0.057	0.216***	0.057	1.241***
x_4_	0.121**	0.062	0.117*	0.062	0.121*	0.062	1.128*
x_5_	0.394***	0.092	0.390***	0.091	0.403***	0.095	1.497***
x_6_	0.241***	0.074	0.241***	0.073	0.221***	0.073	1.247***
x_7_	0.316***	0.085	0.322***	0.085	0.326***	0.089	1.385***
x_8_			0.212	0.157	0.217	0.154	1.243
x_9_			-0.183	0.213	-0.117	0.213	0.889
x_10_					0.608***	0.193	1.837***
constant	-7.661***	0.601	-7.771***	0.930	-8.273***	0.970	0.000***
Log pseudolikelihood	-398.933		-397.723		-392.494		
Wald ch2	(7) 158.71		(9) 160.63		(10) 160.03		
Prob > ch2	0.0000		0.0000		0.0000		
Pseudo R2	0.2043		0.2067		0.2171		

*Note*: Robust standard error in S.E. column.

*** p-Value ≤ 0.01.

** p-Value ≤ 0.05.

* p-Value ≤ 0.1.

**Table 6:** Logistic regression for process innovation.

Variables	Model 1		Model 2		Model 3		
	*β*	S.E.	*β*	S.E.	*β*	S.E.	OR
x_1_	0.289***	0.060	0.290***	0.060	0.291***	0.061	1.338***
x_2_	-0.043	0.058	-0.052	0.059	-0.043	0.058	0.958
x_3_	0.287***	0.059	0.283***	0.059	0.293***	0.059	1.341***
x_4_	-0.006	0.060	-0.004	0.060	-0.004	0.060	0.996
x_5_	0.368***	0.098	0.364***	0.098	0.373***	0.099	1.452***
x_6_	0.229***	0.088	0.251***	0.087	0.237***	0.087	1.268***
x_7_	0.335***	0.084	0.342***	0.085	0.341***	0.086	1.407***
x_8_			0.138	0.162	0.141	0.160	1.151
x_9_			0.337	0.228	0.385*	0.227	1.469*
x_10_					0.445**	0.204	1.560**
constant	7.807***	0.707	-9.305***	1.083	-9.651***	1.112	0.000***
Log pseudolikelihood	-358.395		-356.695		-354.272		
Wald ch2	(7) 106.38		(9) 111.03		(10) 109.86		
Prob > ch2	0.0000		0.0000		0.0000		
Pseudo R2	0.1872		0.1910		0.1965		

*Note*: Robust standard error in S.E. column.

*** p-Value ≤ 0.01.

** p-Value ≤ 0.05.

* p-Value ≤ 0.1.

**Table 7:** Logistic regression for both product and process innovation.

Variables	Model 1		Model 2		Model 3		
	*β*	S.E.	*β*	S.E.	*β*	S.E.	OR
x_1_	0.395***	0.072	0.398***	0.071	0.403***	0.072	1.496***
x_2_	0.006	0.064	0.005	0.064	0.016	0.064	1.016
x_3_	0.304***	0.069	0.310***	0.070	0.324***	0.069	1.383***
x_4_	0.007	0.068	0.005	0.068	0.002	0.068	1.002
x_5_	0.447***	0.118	0.438***	0.116	0.451***	0.120	1.569***
x_6_	0.305***	0.098	0.323***	0.098	0.310***	0.097	1.363***
x_7_	0.410***	0.095	0.423***	0.095	0.427***	0.098	1.532***
x_8_			0.273	0.192	0.274	0.187	1.316
x_9_			0.092	0.251	0.168	0.245	1.183
x_10_					0.600***	0.235	1.821***
constant	-10.050***	0.740	-11.231***	1.175	-11.804***	1.203	0.000
Log pseudolikelihood	-283.912		-282.626		-279.180		
Wald ch2	(7) 158.84		(9) 167.08		(10) 166.44		
Prob > ch2	0.0000		0.0000		0.0000		
Pseudo R2	0.2586		0.2619		0.2709		

*Note*: Robust standard error in S.E. column.

*** p-Value ≤ 0.01.

** p-Value ≤ 0.05.

* p-Value ≤ 0.1.

First, in Tables 5–7, we present the basic model taking into account the variables describing innovation management capability (Model 1). Then, variables describing the characteristics of enterprises are introduced into the estimation (Model 2). As the last one, we estimate the final model with an additionally introduced variable concerning the gender of the entrepreneur—woman (Model 3).

The performed plausibility tests, in each case, indicate the significance of the estimated models. This gives us the basis for further interpretation of the obtained results. It is also worth emphasizing that in each case, the introduction of additional variables improved the parameters of the models (Log pseudolikelihood, Wald chi2 and Pseudo R2) and indicated an improvement in the goodness of fit of the estimations. In the case of the final model (Model 3), we also showed the odds ratios (OR) to interpret the model estimation results.

As can be seen in Tables 5–7, the same six explanatory variables are statistically significant for each of the explained variables (in final Model 3). These are: identifying, generating, assessing and pursuing ideas for innovation (x_1_), allocating resources to innovation activities (x_3_), integrating external knowledge and other external inputs into a firm’s innovation activities (x_5_), monitoring the results of innovation activities and learning from experience (x_6_), exploiting and managing innovations and other knowledge that has been generated as part of a firm’s innovation activities (x_7_) and female gender of entrepreneur (x_10_). It is worth emphasizing, however, that certain differences can be observed between the determinants of product and process innovations. In the case of product innovations, an additional influencing factor turned out to x4—that is managing innovation activities conducted in collaboration with external partners. However, in the case of process innovations, the age of the enterprise is also statistically significant (x_9_), while in the case of product innovations it is not significant.

**Fig 2:**
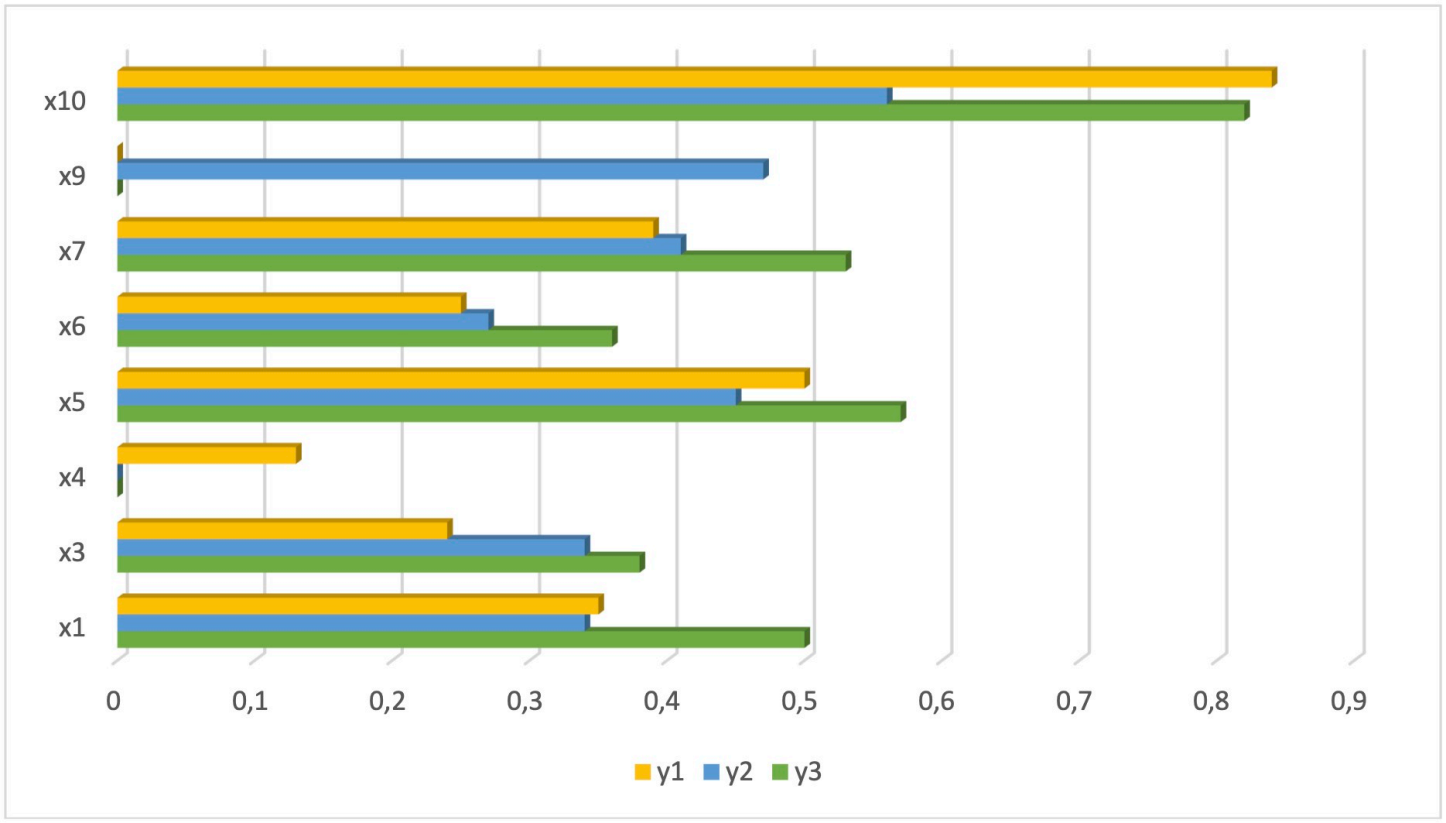
Odds ratio for statistically significant variables for all logistic regression models.

The odds ratios for statistically significant variables for Models 3 (for y1, y2 and y3) are presented in Fig 2.

In the following part, we would like to focus on those small enterprises that have introduced both product and process innovations (y_3_). As can be seen, the parameter estimates took positive signs. In other words, the impact of the explanatory variables included in the model on the explained variable increases the chances of introducing product and process innovations by small enterprises. The explanatory variables that exert a statistically significant effect on the innovativeness of small enterprises, at 0.01 confidence level, include identifying, generating, assessing and pursuing ideas for innovation (x_1_), allocating resources to innovation activities (x_3_), integrating external knowledge and other external inputs into a firm’s innovation activities (x_5_), monitoring the results of innovation activities and learning from experience (x_6_), exploiting and managing innovations and other knowledge that has been generated as part of a firm’s innovation activities (x_7_) and female gender of entrepreneur (x_10_).

## 5. Discussion

The main objective of this study was to analyse the influence of the entrepreneur’s gender—as one of the elements building management capabilities—on the innovation of enterprises. The research focuses on small enterprises that constitute the vast majority of companies in Poland, as well as in most world’s economies. Therefore, we conducted an empirical study based on a sample of 1017 small firms from the Kujawsko-pomorskie region in central-northern Poland, in which we used a questionnaire to obtain data to test three models using statistical procedures and techniques.

We performed the interpretation of the model results based on the odds ratios (OR). And so, in the case of introducing innovations, bearing in mind the *ceteris paribus* assumption—that is, the remaining variables of the model unchanged, it can be concluded that the chance of introducing innovations is greater in the group of small enterprises which are:

- identifying, generating, assessing, and pursuing ideas for innovation: by 34.9% for product innovations, by 33.8% for process innovations, and by 49.6% in total for product and process innovations (x_1_),
- allocating resources to innovation activities: by 24.1% for product innovations, by 34.1% for process innovations, and by 38.3% in total for product and process innovations (x_3_),
- managing innovation activities conducted in collaboration with external partners by 12.8% for product innovations (x_4_),
- integrating external knowledge and other external inputs into a firm’s innovation activities: by 49.7% for product innovations, by 45.2% for process innovations, and by 56.9% in total for product and process innovations (x_5_).
- monitoring the results of innovation activities and learning from experience: by 24.7% for product innovations, by 26.8% for process innovations, and by 36.3% in total for product and process innovations (x_6_).
- exploiting and managing innovations and other knowledge that has been generated as part of a firm’s innovation activities: by 38.5% for product innovations, by 40.7% for process innovations and by 53.2% in total for product and process innovations (x_7_).
- have a longer lifetime by 46.9% for process innovations (x_9_).
- are managed by female entrepreneurs: by 83.7% for product innovations, by 56% for process innovations, and by 82.1% in total for product and process innovations (x_10_).

The results of the study do not allow to conclude that the chances of introducing product and process innovation are greater in the group of small enterprises differentiated by size (x_8_), or which organize innovative activities inside (x_2_).

Our findings suggest that the performance of managerial functions by women increases the chances of introducing product and process innovations, when considered both together and separately, which allows for a positive verification of H_1_ (The entrepreneur’s female gender exerts a positive impact on small business innovativeness). The gender factor in our research increased the chances of introducing all the types of innovations we studied to the greatest extent, which indicates a significant role of gender in innovation processes. Although we obtained results different from Foss et al. showing that the level of innovativeness of women is similar to that of men [78], we believe that our results do not negate those obtained by Foss et al. Explaining the lower level of women’s activity in the field of innovation, Foss et al. indicated the limitations imposed by organizational practices or the lower level of support from their superiors felt by women [78], while our respondents were women with the highest managerial functions, and therefore, in their case, practices and feelings could not play a significant role because they played a key role in their creation. Our observation that in the case of women, greater chances of implementing innovations exist in the group of product innovations (83.7%) than in the group of process innovations (56%), is consistent with the research results achieved by Fernandez, who pointed out that product innovations are related to solving human problems based on interactions between employees and external agents, while process innovations are based on solving technical problems. Consequently, the gender diversity of the R&D team contributes more to product innovation than to process innovation [117].

Almost all of the researched elements of innovation management capability contributed to an increase in the chances of introducing product and process innovations. Only in the case of organizing innovation activities within the companies (x_2_), no impact on product and process innovations was observed both collectively and separately, and no impact of managing innovation activities conducted in collaboration with external partners (x_4_) on the increase in the chances of introducing process innovations was observed. This means that H_2_ (Innovation management capability exerts a positive impact on small business innovativeness) has been partially verified.

The study showed that there is no basis to conclude that organizing innovation activities within the companies increases the chances of introducing innovation (x_2_). Although small enterprises are widely recognized as important innovators and, in the past years, have registered increasing levels of patenting [118], the observed phenomenon can be explained by referring to small business risk aversion [119] or, more likely, it results from the technological advancement of the sectors in which the surveyed small enterprises operated. It can be seen that while high-tech small enterprises are more involved in process and product innovation, non-high-tech small enterprises decide to replace research and development with management practices and cooperation with external partners [120]. Therefore, we believe that the research should be deepened by referring to the influence of technological advancement of small enterprise on the variables analysed.

When attempting to explain the lack of impact of managing innovation activities conducted in collaboration with external partners (x_4_) on the increase in the chances of introducing process innovations, it can be noticed that the results of research to date [82,121] suggest that this type of activities is related to product innovations rather than process innovations, indicating that limited resources force SMEs to refer to business ties and social networks for valuable resources in order to effectively use the opportunities arising from identified customer needs [82].

The results of the study suggest that the observed significance of the impact of innovation management capability on the chances of implementation of all types of innovations analysed by us was similar. The following had the greatest impact in the presented order: (1) x_5_—integrating external knowledge and other external inputs into a firm’s innovation activities; (2) x_7_—exploiting and managing innovations and other knowledge that has been generated as part of a firm’s innovation activities; (3) x_1_—identifying, generating, assessing and pursuing ideas for innovation; (4)–x_3_—allocating resources to innovation activities; (5) x_6_—monitoring the results of innovation activities and learning from experience.

The results of the study show the importance of managing knowledge both acquired from the environment and generated inside the organization. This confirms the postulates of Simeone’s et al. indicating the purposefulness of developing mechanisms that allow to acquire external knowledge, transfer it internally and integrate it with existing resources [122], as well as suggestions made by Subrahmanya relating to the acquisition of external knowledge and skills, allowing to supplement one’s own competences [123]. The relatively low assessment of the impact of allocating resources to innovation activities is puzzling, which most likely results from the negative impact of financial constraints on enterprises’ decisions regarding involvement in innovative activities, as indicated by Ayalew and Xianzhi [124].

The results of the study do not allow for the conclusion of the existence of premises confirming the correctness of the hypothesis H_3_ (Firm characteristics exert a positive impact on small business innovativeness). This does not mean that this relationship does not exist, but it seems to us that it is indirect. In other words, neither age nor size translates directly into an increase in process and product innovation (only in relation to process innovations, the age affects the increase in the chances of their introduction at 0.1 confidence level). The above observation is important as it forces the need to search for factors moderating innovation in terms of age and size, which should be the subject of further research in this area.

## 6. Conclusion

In this article, we dealt with the issue of the influence of the entrepreneur’s gender as one of the elements building management capabilities on product and process innovation in small enterprises, which is insufficiently described in the literature. In attempting to outline the scope of the research, we adopted the approach proposed in the Oslo Manual, pointing to innovation management capability as one of the five elements building management capabilities [11].

In an attempt to indicate which variables, increase the chances of implementing product and process innovations, in addition to the seven variables constituting innovation management capability, we also took into account the characteristics of the organization and gender, putting forward the following three hypotheses:

*H*_*1*_: *The entrepreneur’s female gender exerts a positive impact on small business innovativeness*,
*H*_*2*_: *Innovation management capability exerts a positive impact on small business innovativeness*,
*H*_*3*_: *Firm characteristics exert a positive impact on small business innovativeness*.

The results of the study suggest that the female gender of the entrepreneur has a positive impact on the product and process innovation of small enterprises, because the chances of introducing product innovation are higher by 83.7%, process innovation by 56% and product and process innovation together by 82.1% in the group of small enterprises managed by female entrepreneurs, which confirms the correctness of the first research hypothesis. The results of the study also partially confirmed the correctness of the second hypothesis, according to which innovation management capability exerts a positive impact on small business innovativeness (the dependence was not observed only in relation to the impact of organizing innovation activities within the firm (x_2_) on the increase in the chances of introducing product and process innovations, both jointly and separately, and managing innovation activities conducted in collaboration with external partners (x_4_) on the increase in the chances of introducing process innovations and both product and process), and did not allow to confirm the hypothesis relating to the impact of the company’s characteristics—size (x_8_) and age (x_9_) on the increase in the chances of introducing product and process innovations (age it only influenced the chances of introducing process innovations).

The results of our research contribute to the practice and theory of management. In the case of the contribution to practice, the findings highlight the role of female entrepreneurs in the processes of implementing innovation activities. When it comes to contributing to theory, we have shown that product and process innovations are more likely to be implemented where the company is managed by a female entrepreneur, which points to the importance of gender as a determinant of process and product innovations.

Like most empirical studies, this research has certain limitations which constrain its generalizability. We are aware of these limitations, which, however, they provide opportunities for future research. First, our sample is composed only of small enterprises, so it is difficult to generalize the findings to all types of enterprises. Second, research was carried out for all sectors without differentiating between them. We believe that it would be worthwhile to conduct research separately in traditional and modern sectors to check how the studied variables behave and whether in any of the sector’s gender does not cease to be an important factor increasing the chances of introducing product and process innovations. Third, subjective rather than objective data was used to measure innovation management capability and the innovativeness. In future research, it would be worth to use the objective data of measure innovation management capability and the innovativeness. Fourth, given the irregular nature of innovation, our snapshot survey may not have captured significant long-term trends. Therefore, we believe it is worth carrying out longitudinal studies that would allow us to show whether the dependence we have identified persists. Fifth, we studied the innovation of female entrepreneurs, not differentiating the roles of female owners and female managers, while the results of Dohse, Goel, and Nelson studies show that female owners, rather than female managers, are more likely to introduce innovations [103]. Therefore, in the future studies it would be worth examining innovative behaviours by differentiating female owners and female managers, adding, due to the specificity of small business, the category of female owners-managers.
